# Early penile prosthesis infections in the contemporary antimicrobial era: Incidence and predictive factors from a regional cohort

**DOI:** 10.1002/bco2.70235

**Published:** 2026-06-26

**Authors:** Imaad Amanulla, Mohamed Mubarak, John Hayes, Michael Rafeh, Ethan Clifford, Jon Barclay, Trevor Dorkin, Anthony Emmanuel

**Affiliations:** ^1^ Department of Urology Freeman Hospital, Newcastle upon Tyne Hospitals NHS Foundation Trust Newcastle upon Tyne UK; ^2^ Biosciences Institute, Faculty of Medical Sciences Newcastle University Newcastle upon Tyne UK

**Keywords:** diabetes mellitus, erectile dysfunction, immunosuppression, penile prosthesis, post‐operative infection, prosthetic device complications, prosthetic infection

## Abstract

**Objectives:**

To evaluate the incidence and predictive factors of early penile prosthesis (PP) infection within a contemporary regional cohort in the North of England.

**Patients and methods:**

A retrospective review of all PP implantations was conducted at a tertiary urology and andrology centre between 2018 and 2025, with variables including age, BMI, smoking, relevant comorbidities, pre‐operative HbA1C, immunosuppressant use and device‐related variables. Data were analysed using SPSS v29, with univariate logistic regression performed to identify factors of early infection (≤30 days). A *p*‐value < 0.05 was considered statistically significant.

**Results:**

A total of 103 patients underwent PP implantations. Mean age at implant was 59.4 ± 9.9 years. The comorbidity burden of diabetes, hypertension and dyslipidaemia was 35.0%, 49.5% and 39.8%, respectively, with mean pre‐operative HbA1C at 45.6 mmol/mol. Notably, 9.7% of patients were on chronic immunosuppressive therapy. Early prosthetic infection was identified in six patients (5.8%). On univariate analysis, there was a strong association between immunosuppressant use and infection (50.0% vs 7.2%, *p* = 0.02; OR 12.0, 95% CI 1.7–85.0). The variables of age, BMI, diabetes, hypertension, dyslipidaemia, HbA1C and implant type were not associated with infection.

**Conclusion:**

In this contemporary regional study, early PP infection was identified in 5.8% of the cohort, with chronic immunosuppressant use being the dominant predictor of infection. Other metabolic, clinical and implant factors were not predictive of infection. This demonstrates immunosuppression being a persistent biological vulnerability requiring risk stratification and targeted perioperative strategies. Larger, prospective multicentre studies are required to further validate its impact and inform evidence‐based infection‐prevention protocols.

## 1 INTRODUCTION

Erectile dysfunction (ED) is defined as the inability to achieve or maintain a rigid penile erection sufficient for sexual intercourse.^1^ It is a prevalent condition affecting between 3% and 76.5% of men worldwide with an increasing incidence concordant with the rising burden of metabolic and cardiovascular disease^1^

For men who fail or decline conservative or pharmacological therapies, penile prosthesis is the definitive surgical management offering high rates of long‐term functional success and patient satisfaction.^2^, ^3^ Despite advances in prosthesis technology and peri‐operative anti‐microbial prophylaxis, device infection accounts for 1%–3% of complications.^2^ Infections often necessitate salvage procedures, explantation and delayed re‐implantation, which in turn compounds the problem of ED with significant physical, psychological and economic consequences.^2^

Prosthetic infection has a multifactorial pathogenesis, though it is primarily thought to originate from bacterial adhesion and overgrowth on implant surfaces, which in turn confer resistance to host immune responses.^3^, ^4^ The growing issue of antimicrobial resistance has added a further barrier to treating these infections, which further complicate preventive strategies.^5^ Numerous patient and procedure related factors have been reported in the literature to be associated with penile prosthetic infection. Carvajal et al. highlight diabetes and immunosuppression as significant risk factors, though obesity and drug addiction are reported to contribute.^3^ Protective procedural factors include peri‐operative antimicrobial strategies, infection‐retardant implant coatings and variations of intra‐operative antiseptic irrigation and solutions.^3^ The use of intraoperative washes such as Irrisept solution (0.05% chlorhexidine gluconate) has heterogeneous evidence of effectiveness, with the literature demonstrating effectiveness against modern‐day organisms when compared with normal saline yet, also reporting Irrisept to be inferior when compared to antibiotic irrigants and increasing risk of infection in hydrophilic penile prostheses.^6^, ^7^, ^8^, ^9^

The relative contributions of these factors to penile prosthetic infection risk overall remain unclear, with most data derived from heterogeneous populations, highlighting the need for contemporary data and standardised guidelines to risk‐stratify patients accordingly.

Consequently, contemporary, region‐specific data are required to better characterise infection risk and inform patient selection and peri‐operative optimisation strategies. This study evaluates the incidence and predictive factors of early penile prosthetic infection within a contemporary regional cohort in the North of England.

## 2 PATIENTS AND METHODS

A retrospective review was conducted of all patients who underwent penile prosthesis implantation at a single regional tertiary urology and andrology centre between 2018 and 2025. Consecutive cases, which included both primary and revision implantations, were identified using electronic medical records. Overall, the andrology service at our centre follows a comprehensive and structured pathway. This includes initial counselling in clinic, further pre‐prosthesis implantation counselling with an andrology nurse specialist, discussion at the andrology multidisciplinary team meeting and targeted imaging for complex cases. Our standard antimicrobial prophylaxis regimen comprises teicoplanin, gentamicin and metronidazole for all patients. Fluconazole is added for patients with diabetes, those who are immunosuppressed and in cases of prosthesis revision. Implants used include Boston Scientific AMS 700 with inhibizone and Coloplast implants soaked in rifampicin and gentamicin.

Baseline demographic and clinical characteristics that were extracted included age at surgery, body mass index (BMI), smoking status, relevant comorbidities (diabetes, hypertension, dyslipidaemia, ischaemic heart disease, Peyronie's disease, history of radical prostatectomy/cystectomy/pelvic surgery, prior pelvic radiotherapy), steroid therapy and immunosuppressant therapy including infliximab, sulfasalizine tacrolimus and mycofenolate mofetil, indicated for conditions such as Crohn's disease, adrenal insufficiency and post‐organ transplant. Implant‐related variables included type of implant: inflatable or malleable. Pre‐operative glycaemic control was assessed using HbA1c levels done at pre‐assessment prior to surgery in mmol/mol as per local practices. The primary outcome of interest was defined as clinically diagnosed penile prosthetic device infection occurring within 90 days of implantation, requiring medical or surgical intervention.

Data were anonymised and collated in Microsoft Excel, with subsequent statistical analysis done using SPSS v29 (IBM Corp., Armonk, NY, USA). Continuous variables were summarised using means and standard deviations. Univariate logistic regression analyses were performed to evaluate associations between patient and procedure‐related factors and the risk of early infection. Results were expressed as odds ratios (ORs) with 95% confidence intervals (95% CI). A *p*‐value < 0.05 was considered statistically significant.

## 3 RESULTS

A total of 103 patients underwent penile prosthesis implantations between 2018 and 2025, with a mean age of 59.4 ± 9.9 years at implant and mean BMI of 29.7 ± 4.7 kg/m^2^. The respective comorbidity burden included diabetes mellitus in 35.0% of patients, hypertension in 49.5% and dyslipidaemia in 39.8%. Notably, 9.7% of patients were on chronic immunosuppressive therapy. Of note, all patients continued routine use of their immunosuppressive therapy peri‐operatively. When evaluating implant characteristics, it was evident that 79.6% of patients underwent inflatable penile prosthetic insertion, while 20.4% received malleable implants. Implant brands used included AMS/Boston (46.6%) and Coloplast (49.5%). Pre‐operative HbA1C measurements were available for 91 patients, with a mean value of 45.6 ± 12.3 mmol/mol. A summary of proportions and total numbers can be viewed in Table 1.

**TABLE 1:** Baseline demographics and clinical characteristics of patients undergoing penile prosthesis implantation.

Variable	Overall (*n* = 103)	Infected (*n* = 6)	Non‐infected (*n* = 97)	*p*‐value
Age (years), mean ± SD	59.4 ± 9.9	59.5 ± 8.4	59.4 ± 10.0	0.97
BMI (kg/m^2^), mean ± SD	29.7 ± 4.7	29.5 ± 2.7	29.7 ± 4.8	0.92
Diabetes mellitus, *n* (%)	36 (35.0)	2 (33.3)	34 (35.1)	0.93
Hypertension, *n* (%)	51 (49.5)	2 (33.3)	49 (50.5)	0.45
Dyslipidaemia, *n* (%)	41 (39.8)	2 (33.3)	39 (40.2)	0.75
Ischaemic heart disease, *n* (%)	28 (27.2)	2 (33.3)	26 (26.8)	0.71
Steroid use, *n* (%)	7 (6.8)	1 (16.7)	6 (6.2)	0.30
Immunosuppressive therapy, *n* (%)	10 (9.7)	3 (50.0)	7 (7.2)	0.02
HbA1c (mmol/mol), mean ± SD	45.6 ± 12.3	46.6 ± 11.4	45.5 ± 12.4	0.81
Smoking, *n* (%)	19 (18.4)	1 (16.7)	18 (18.6)	0.89

Early penile prosthesis infection within 90 days of implant occurred in six patients (5.8%). Out of these six patients, four patients developed early penile prosthesis infection within 30 days of implant. Baseline demographic and metabolic characteristics were comparable between infected and non‐infected groups: age (59.5 ± 8.4 vs. 59.4 ± 10.0 years), BMI (29.5 ± 2.7 vs. 29.7 ± 4.8 kg/m^2^) and HbA1c (46.6 ± 11.4 vs. 45.5 ± 12.4 mmol/mol). The prevalence of diabetes (33.3% vs. 35.1%), hypertension (33.3% vs. 50.5%) and dyslipidaemia (33.3% vs. 40.2%) did not differ significantly between infected and non‐infected groups.

On univariate logistic regression analysis, immunosuppressive therapy was strongly associated with early infection (50.0% vs. 7.2%, *p* = 0.02; OR 12.0, 95% CI 1.7–85.0). No significant associations were identified for age, BMI, diabetes mellitus, hypertension, dyslipidaemia, pre‐operative HbA1c or implant type (*p* > 0.05).

The mean time to infection was 22.2 days with no statistically significant difference observed between immunocompromised (19.7 days) and non‐immunocompromised patients (24.7 days).

When evaluating if immunosuppressant therapy were stopped intra‐operatively, none of the immunosuppressants had been stopped in infected cases. Organisms isolated from infected patients included *Staphylococcus lugdunensis*, *Staphylococcus Epidermidis*, *Pseudomonas aeruginosa*, *Candida glabrata* and *Klebsiella* species. There was no clear difference in isolated organisms noted between immunosuppressed and non‐immunosuppressed groups. Mean time to infection and respective organisms isolated in patients who had infected prostheses are included in Table 2.

**TABLE 2:** Time to infection and isolated organisms in patients with infected penile prostheses.

Patient	Time to infection (days)	Organism isolated
1	46	*Staphylococcus lugdunensis* and *Staphylococcus epidermidis*
2	28	No growth
3	32	*Pseudomonas aeruginosa*
4	3	No growth
5	14	*Candida glabrata*
6	10	Klebsiella

## 4 DISCUSSION

Penile prosthesis infection remains a serious complication in prosthetic urology in contemporary practice.^10^ Despite modern antimicrobial practices, implant infection creates a multifaceted impact on patients and the wider economy.^3^, ^10^ Implant infection, in severe cases, could require removal of the prosthesis with difficult subsequent revision due to scarring of the corpus cavernosum of the penis.^11^ The associated economic impact is considerable, driven by prolonged hospitalisation, increased antimicrobial use and revision implant cost.^11^

In interpreting these findings, two key observations emerge from our cohort. First, the early penile prosthesis infection rate was 5.8%, consistent with modern series.^2^, ^12^ Second, chronic immunosuppression emerged as the dominant predictor of early infection, whereas metabolic factors were not associated with increased risk.

At a population level, our reported findings align with contemporary large‐scale series and meta‐analyses.^2^, ^3^ In a study evaluating infection rates among 18 475 males who had inflatable penile prostheses, 3.1% developed infection at 3 years, with 2.5% occurring within the first 6 months, with the most notable predictors being type 2 diabetes mellitus, spinal cord injury and obesity.^2^ In a large systematic review and meta‐analysis encompassing 175 592 patients, Carvajal et al. reported a significantly increased risk of penile prosthesis infection associated with diabetes mellitus, immunosuppression and obesity,^3^ which aligns with our study in terms of immunosuppression being a predictor but contrasts with diabetes being a predictor. Likewise, Gon et al. further highlighted the association between diabetes and prosthetic infection, which contrasts with our findings.^11^ Recent meta‐analyses, including that by Corona et al. (k = 11, *n* = 10 024), Menshchikov et al. (*n* = 271 226) and Sim et al. (*n* = 6964), further suggest that poor glycaemic control, most notably HbA1c levels 7.5% or higher, is associated with increased infection risk at a population level.^13^, ^14^, ^15^ Despite this, the greater evidence remains contradictory as other studies, such as that by Nijhar et al. and Osman et al., demonstrated that diabetes and higher HbA1c levels did not increase the risk of prosthetic infection.^16^, ^17^

Taken together, these findings suggest that metabolic risk may be modifiable through peri‐operative optimisation, whereas immunosuppression represents a persistent biological vulnerability requiring targeted strategies. However, evidence linking immunosuppression to penile prosthesis infection remains heterogeneous. Carvajal et al. demonstrated substantially higher penile prosthetic infection risk in immunosuppressed patients, though the same review found no association between organ transplant and infection.^3^ A study by Sidi et al. demonstrated no association between immunosuppressed transplant patients and prosthetic infection.^18^ A further study comparing 245 immunocompromised patients (on immunosuppressants including steroids, methotrexate and biologics), with 705 immunocompetent patients, found no difference in re‐operation rates due to infection.^19^ The evident contrast in various predictive factors could be the result of patient case‐mix and regional practice patterns, underscoring the importance of risk stratification and tailored peri‐operative approaches in contemporary practice.

Pertinent negatives in terms of predictors of infection include age, BMI and smoking, as these factors were comparable in infected and non‐infected groups. Age having no effect on risk for prosthetic infections aligns with international literature.^20^ There is mixed evidence when it comes to the effect of BMI on prosthetic infection, with some studies such as that by Wilson et al. and Hebert et al. demonstrating no correlation between BMI and prosthetic infection, while others such as that by Brant et al. present obesity as a predictive prosthetic infection risk.^2^, ^20^, ^21^ The effect of smoking on prosthetic infection remains heterogeneous. Wilson et al. present smoking as a risk factor for glans necrosis following penile prosthesis implantation.^21^ However, a retrospective review by Cakan et al. stated smoking was not a risk factor.^22^

Penile prosthesis infection persists despite contemporary antimicrobial strategies due to its multifactorial pathogenesis, including microbial colonisation at the time of implantation, and the presence of more virulent microorganisms including fungal species.^3^, ^23^ In our cohort, the absence of a significant association between diabetes and prosthetic infection likely reflects strict patient selection that is applied across our trust, with adherence to anaesthesic guidelines that patients should have their procedure deferred if HbA1C is >70 mmol/mol (8.5%).^24^ This approach mitigates the effects of peri‐operative hyperglycaemia on immune function and wound healing, thus reducing the risk of post‐operative infection. In contrast, immunosuppressed patients remained at increased risk of early infection, suggesting that metabolic optimisation alone is insufficient to fully mitigate infection risk in this subgroup. This underscores the need for respective identification and meticulous optimisation of immunocompromised patients prior to penile prosthesis implantation through a multidisciplinary approach. This may include liaising with respective teams including those involved in transplant patients to ensure immunosuppression is revised in a manner that poses the lowest risk of infection. By optimising these peri‐operative strategies, the clinical, psychological and economic burden of infections can be reduced.^3^, ^11^

This study provides valuable real‐world insight into penile prosthetic infection outcomes within the north‐east of England, a geographically and demographically diverse region for which contemporary data are lacking. However, several limitations merit consideration. The retrospective single‐centre design limits causal inference and may introduce unmeasured confounding. The modest cohort size and low event rate constrain statistical power. Future work should focus on multicentre collaboration across the region and nationally, with the development of a prospective UK penile prosthesis registry analogous to the Prospective Urologic Multi‐centre Penile Prosthesis (PUMP) database.^9^

Early infection following penile prosthesis surgery remains uncommon in the contemporary antimicrobial era. In this regional cohort, chronic immunosuppressive therapy emerged as the sole significant predictor of early infection, while metabolic and procedural factors were not associated with increased risk. This highlights the need for targeted peri‐operative planning in this vulnerable patient cohort to optimise surgical outcomes and larger prospective multicentre studies both regionally and nationally to further define modifiable infection determinants and inform evidence‐based risk stratification strategies.

## AUTHOR CONTRIBUTIONS

*Study concept and design*: Mohamed Mubarak, Anthony Emmanuel. *Data acquisition*: Imaad Amanulla, Mohamed Mubarak, John Hayes, Michael Rafeh, Ethan Clifford. *Data analysis and interpretation*: Imaad Amanulla, Mohamed Mubarak, Anthony Emmanuel. *Statistical analysis*: Imaad Amanulla, Mohamed Mubarak. *Drafting of manuscript*: Imaad Amanulla, Mohamed Mubarak. *Critical revision of manuscript*: All Authors.

## CONFLICT OF INTEREST STATEMENT

No conflicts exist.

## ETHICAL STATEMENT

The study was conducted in line with the principles of the Declaration of Helsinki. Given the retrospective nature of the study and the use of anonymised data, the requirement for patient consent was waived. The data included in this study were handled in line with local data protection regulations and policies.
